# Web alert: Microbial electrochemistry for biotechnology

**DOI:** 10.1111/1751-7915.14237

**Published:** 2023-02-23

**Authors:** Lawrence P. Wackett

**Affiliations:** ^1^ Department of Biochemistry, Molecular Biology & Biophysics, BioTechnology Institute University of Minnesota St. Paul Minnesota USA

Microbial electrochemical systems

https://www.frontiersin.org/articles/10.3389/fenvs.2020.00044/full

This systematic review article provides a broad and historical description of microbial electrochemical systems.

Enzyme and microbial electrochemistry

https://pubs.acs.org/doi/10.1021/acsmeasuresciau.2c00042

This tutorial review focuses on the interactions between enzymes, microbes and electrodes. It also covers electroanalytical methods.

Microbial fuel cells

https://www.sciencedirect.com/topics/biochemistry‐genetics‐and‐molecular‐biology/microbial‐fuel‐cell

This page contains a compendium of journal articles, books and book chapters dealing with microbial fuel cells.

Microbially‐based green electronics

https://www.sciencedirect.com/science/article/pii/S0167779920303309

Some microbes produce nanometer‐diameter protein strands that conduct electricity. This report describes their being examined for potential incorporation into electronic devices.

Bioelectrosynthesis

https://link.springer.com/book/10.1007/978‐3‐030‐03299‐9

The link is to a book on electrochemical biosensors, biosynthesis, bioleaching, artificial photosynthesis and other related topics.

Electrobiotechnology

https://dechema‐dfi.de/en/Topics+_+Projects/Electro+biotechnology.html

This site describes a large number of research projects related to the field of electro biotechnology.

Microbial electrochemical technologies

https://www.water.imdea.org/scientific‐infrastructure/pilot‐plants/microbial‐electrochemical‐technologies

This page describes lab scale and field bioreactors that use microbial electrochemistry for projects such as wastewater treatment and the removal of pollutants.

Increasing electrical conductivity in bacteria

https://phys.org/news/2023‐01‐bacteria‐generated‐nanowires‐soil‐oceans.html

This publication describes bacteria that transmit current through protein nanowires and how a dramatic increase in conductivity can be generated by light.

Microbial electrochemistry terminology

https://citeseerx.ist.psu.edu/document?repid=rep1&type=pdf&doi=4704024707debc15e61af0f60800ef8664c639d1

This article introduces terminology and classification systems that derives from the interfacing of microbiology and electrochemistry.

The International Society for Microbial Electrochemistry and Technology (ISMET)

https://is‐met.org

ISMET is an international society that links researchers working in different fields of science and engineering to consider questions related to microorganisms and electrodes.

Microbial electrochemistry for bioremediation

https://www.electra.site/wp‐content/uploads/2020/06/No.6‐paper.pdf

This article provides a review of biolectrochemical systems that are focused on bioremediation applications.

